# Hemolytic Uremic Syndrome Complicated by Severe Neuropsychiatric Symptoms: A Case Report and Review of the Literature

**DOI:** 10.7759/cureus.98610

**Published:** 2025-12-06

**Authors:** Dongze Wang, Wenyuan Wang, Yanyong Wang

**Affiliations:** 1 Department of Neurology, The First Hospital of Hebei Medical University, Shijiazhuang, CHN

**Keywords:** case report, compound chlorzoxazone, drug-induced adverse reaction, hemolytic uremic syndrome, neuropsychiatric manifestations, thrombotic microangiopathy

## Abstract

Hemolytic uremic syndrome (HUS) is a thrombotic microangiopathy (TMA) characterized by microangiopathic hemolytic anemia, thrombocytopenia, and acute kidney injury (AKI). Adult-onset cases that initially manifest with predominantly neuropsychiatric symptoms are uncommon and may delay diagnosis. We describe the case of a 57-year-old female who developed acute cognitive decline, psychomotor retardation, and tremor following the recent use of a compound chlorzoxazone preparation. Laboratory evaluation revealed thrombocytopenia, hemolysis, and renal impairment, while imaging demonstrated reversible white matter abnormalities. Infectious causes and thrombotic thrombocytopenic purpura (TTP) were excluded, and persistently low complement C3 levels supported complement activation. The clinical course and Naranjo probability score suggested a probable association between drug exposure and HUS. The patient improved with comprehensive supportive management, without complement inhibitor therapy. This report highlights the diagnostic challenge of HUS with predominant neuropsychiatric manifestations and underscores the importance of maintaining a high index of suspicion for TMA in patients with unexplained neurological symptoms and concurrent renal or hematologic abnormalities.

## Introduction

Hemolytic uremic syndrome (HUS) was first described by Gasser et al. in 1955 and is defined by the triad of microangiopathic hemolytic anemia, thrombocytopenia, and acute kidney injury (AKI). It is a leading cause of acute renal failure in children. Most cases are associated with Shiga toxin-producing Escherichia coli (STEC) infection and are classified as typical HUS. In contrast, cases triggered by complement dysregulation, medications, transplantation, or other secondary factors are classified as atypical HUS (aHUS) [1]. Beyond hematologic and renal involvement, HUS can affect multiple organs, with the central nervous system (CNS) often severely impacted [2]. Approximately 20-50% of patients develop neurological manifestations, such as headache, altered consciousness, seizures, hemiparesis, ataxia, or coma, which can be life-threatening if accompanied by cerebral edema, hemorrhage, or ischemic encephalopathy [3].

Drug-induced HUS represents a distinct subset of atypical HUS and is primarily reported in adults. It is most commonly associated with medications such as quinine, calcineurin inhibitors, chemotherapeutic agents, and certain anti-angiogenic drugs, acting through immune-mediated or toxicity-dependent endothelial injury. Although uncommon, drug-induced HUS accounts for a notable proportion of secondary thrombotic microangiopathies (TMAs) in adults and often presents with nonspecific neurological or renal manifestations that complicate early recognition. Given its rarity and the wide variety of implicated agents, timely identification requires careful medication review and exclusion of infectious and hereditary etiologies. This framework is particularly relevant in adult patients with atypical presentations, as in the present case.

Although HUS and aHUS share overlapping clinical features and are challenging to differentiate via routine tests, their mechanisms and treatments differ. Typical HUS involves toxin-mediated endothelial injury and microvascular thrombosis, whereas aHUS stems from uncontrolled complement activation [4]. Identifying the etiology is vital for appropriate therapy and prognosis, especially in cases with neurological involvement.

We report a rare case of drug-induced HUS in an adult patient presenting initially with acute neuropsychiatric symptoms. Reports describing drug-induced HUS with predominant neuropsychiatric manifestations in adults remain exceedingly scarce. Through detailed clinical observation, laboratory evaluation, and literature review, we highlight diagnostic challenges and management strategies to aid early recognition and evidence-based treatment of this uncommon condition.

## Case presentation

Clinical data

A 57-year-old female (height, 165 cm; weight not recorded due to prolonged bed rest) was admitted to the Department of Psychosomatic Medicine at The First Hospital of Hebei Medical University on June 9, 2025, with a seven-day history of insomnia and fatigue accompanied by hand tremor and slurred speech. She had recently been diagnosed with type 2 diabetes mellitus and reported significant fear, anxiety, and worry about her illness. Before admission, she had experienced abdominal pain but denied diarrhea. Her medication history included the use of a compound chlorzoxazone preparation. However, the exact duration, dosing, and timing of the final dose could not be reliably determined due to a lack of documented outpatient records and impaired recall at the time of presentation.

**Figure 1:**
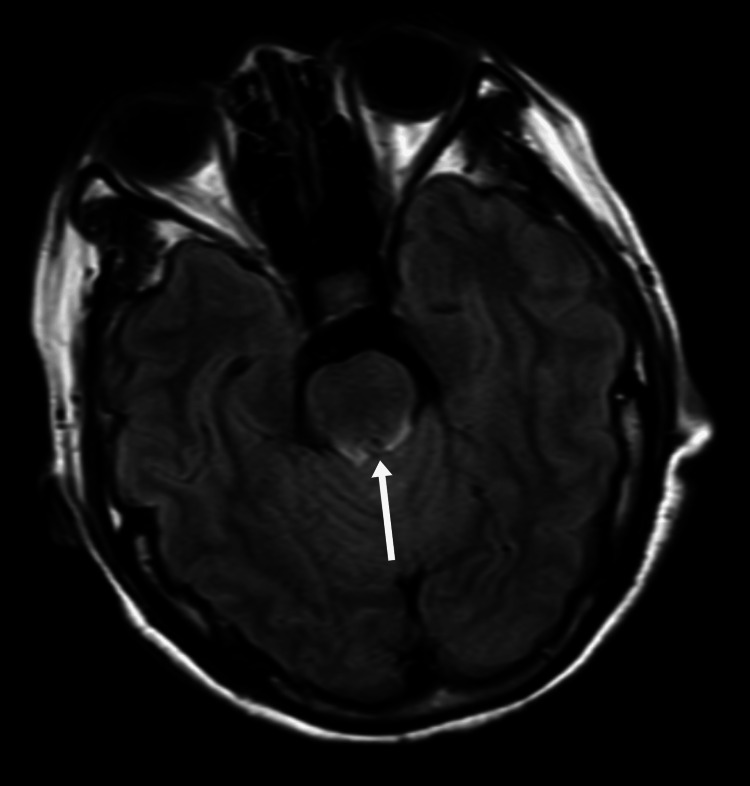
Axial FLAIR MRI of the cerebral peduncle before treatment As indicated by the white arrow, there is an abnormally high signal in the cerebral peduncle FLAIR: fluid-attenuated inversion recovery; MRI: magnetic resonance imaging

**Figure 2:**
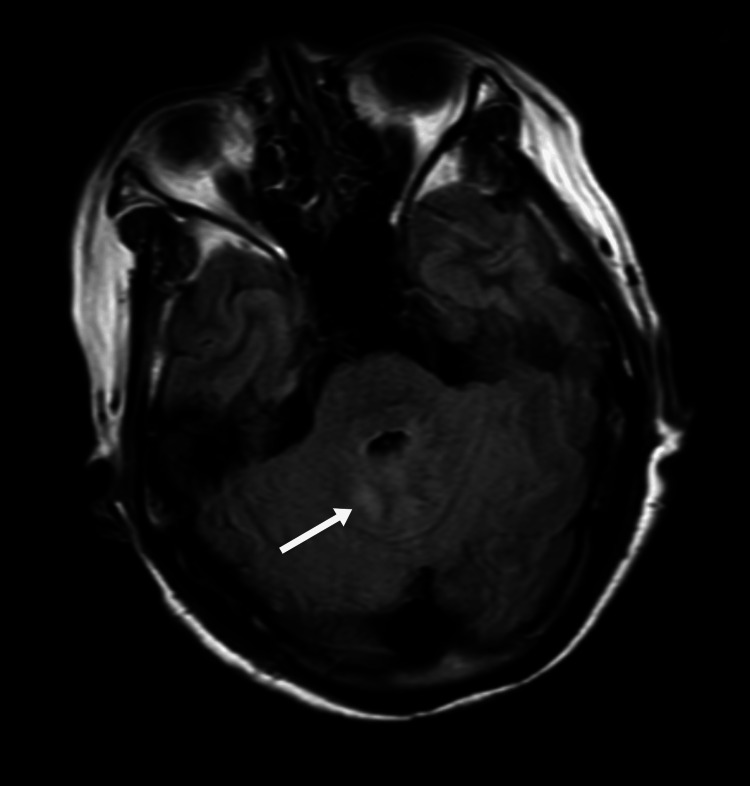
Axial FLAIR MRI of the cerebellar dentate nucleus before treatment As indicated by the white arrow, there is an abnormally high signal in the bilateral symmetric dentate nuclei of the cerebellum FLAIR: fluid-attenuated inversion recovery; MRI: magnetic resonance imaging

On admission, her vital signs were stable: temperature: 36.5°C, pulse: 86 beats/min, respiratory rate: 18 breaths/min, and blood pressure: 124/85 mmHg. Head CT revealed multiple lacunar lesions in the bilateral basal ganglia and corona radiata. Brain MRI showed vascular-origin white matter hyperintensities in the bilateral corona radiata, left thalamus, and basal ganglia, with an encephalomalacic focus in the right basal ganglia (Figures 1-2). Urinalysis indicated proteinuria (3+), glycosuria (3+), and markedly elevated urinary microalbumin (8732.5 mg/L). Serial serum creatinine measurements and eGFR calculations were not available due to incomplete laboratory documentation during admission. D-dimer was 0.82 μg/mL. Stool testing showed weakly positive occult blood but was otherwise unremarkable. Complete blood count, liver and renal function, cardiac enzymes, homocysteine, lipid profile, and thyroid function were normal. Quantification of chlorzoxazone or its metabolites was not performed, as such assays are not available in our clinical laboratory and are not part of standard diagnostic evaluation for suspected drug-associated TMA. Initial diagnoses were as follows: (1) anxiety disorder, (2) type 2 diabetes mellitus, (3) grade 2 hypertension (very high risk), (4) cerebral infarction recovery phase, (5) autonomic dysfunction with somatic symptoms, (6) insomnia, (7) tremor, (8) fatigue, (9) loss of appetite, and (10) post-hysterectomy status.

Main treatment course

Early Evaluation and Supportive Management (Day One to Day Two)

During the early phase, the patient presented with depressive mood, insomnia, and fatigue, accompanied by abdominal pain. Although no focal neurological deficits were noted, findings suggested a condition more complex than a purely psychosomatic disorder. Marked proteinuria, elevated urinary microalbumin, and increased D-dimer indicated fibrinolytic activation. By day two, renal dysfunction worsened, with elevated urea (9.6 mmol/L), proteinuria (4+), hematuria (3+; 67 RBCs/μL), and persistent glycosuria (3+; HbA1c: 6.9%), indicating poor glycemic control. Electrolyte imbalance was also evident (hyponatremia, 133 mmol/L; hypokalemia, 3.41 mmol/L). These abnormalities hinted at an evolving AKI. Potassium supplementation, blood pressure optimization, and consultations with endocrinology and nephrology were initiated.

Rapid Deterioration and Formation of the Classic Triad (Day Three to Day Five)

**Figure 3:**
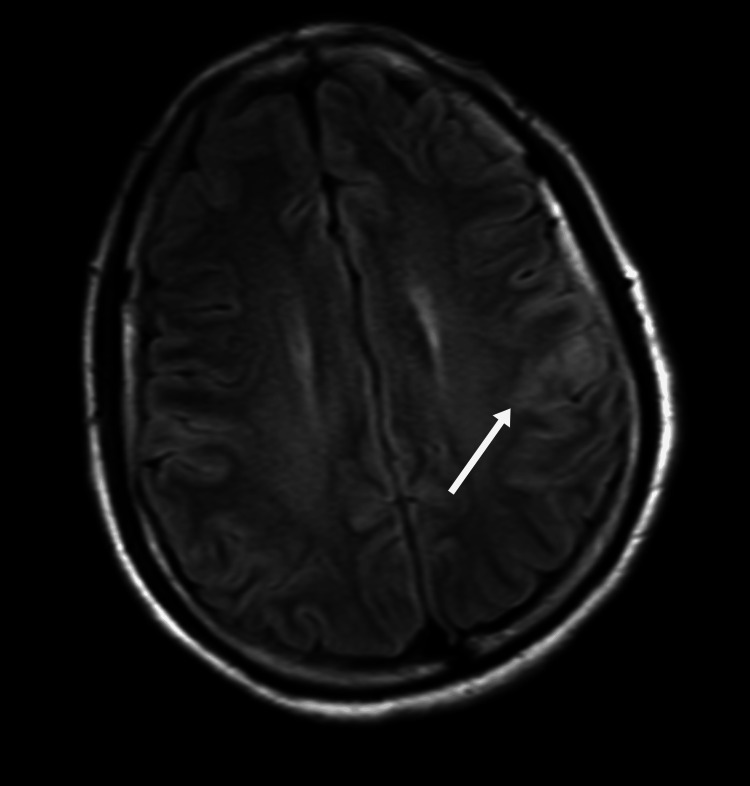
Axial FLAIR MRI showing a hyperintense lesion in the subcortical white matter of the right parietal lobe As indicated by the white arrow, there is a high-signal lesion in the subcortical white matter of the right parietal lobe FLAIR: fluid-attenuated inversion recovery; MRI: magnetic resonance imaging

On day 3, neurological status deteriorated, with psychomotor retardation, reduced speech, and impaired memory. Neurological examination showed increased muscle tone, a positive Babinski sign on the left, and neck stiffness. Electroencephalography (EEG) performed on the same day was normal. Objective cognitive testing demonstrated severe global impairment, with a Mini-Mental State Examination (MMSE) score of 6/30 and a Montreal Cognitive Assessment (MoCA) score of 2/30, indicating profound deficits in attention, orientation, memory, and executive function. Laboratory results showed a sudden drop in platelet count to 41 × 10⁹/L, D-dimer elevation to 1.92 mg/L, and decreased complement C3 (0.87 g/L). The combination of thrombocytopenia, AKI, and neurological symptoms strongly suggested HUS. On day four, lactate dehydrogenase (LDH) rose to 817.7 U/L, with neutrophilia (85.8%), indicating microangiopathic hemolytic anemia (MAHA) and progression of TMA. On day five, haptoglobin dropped below 0.2 g/L, reticulocytes rose to 3.96%, LDH remained elevated (772.1 U/L), and hemoglobin decreased to 104 g/L, confirming the triad of hemolysis, thrombocytopenia, and AKI. Complement C3 remained low (0.80 g/L). MRI showed abnormal signals in the cerebral peduncle, bilateral cerebellar dentate nuclei, and parietal cortices (Figure 3). Although a renal biopsy was not performed, the complement test results heightened the suspicion of atypical HUS.

Multidisciplinary Consultation and Complications (Day Six to Day Seven)

By day six, gastrointestinal bleeding, subcutaneous hemorrhage, and hemoglobinuria developed (hemoglobin, 93 g/L; reticulocytes, 4.71%; platelets, 58 × 10⁹/L), indicating ongoing hemolysis and bleeding tendency. A multidisciplinary team (MDT) meeting was held on June 16 involving nephrology, hematology, intensive care, infectious disease, and radiology, which confirmed TMA with a low PLASMIC score (low thrombocytopenic purpura (TTP) likelihood) and favored HUS. Platelet transfusion was contraindicated. Further work-up included ADAMTS13 activity, pathogen screening, and complement testing. Supportive management continued, focusing on hemodynamic and electrolyte stability, nutrition, psychological care, and prevention of complications.

Diagnostic Refinement and Etiological Focus (Day Eight to Day Nine)

On day 8, hemolysis persisted (reticulocytes, 6.36%; hemoglobin, 87 g/L; D-dimer, 7.09 mg/L). Stool cultures and nucleic acid tests for Salmonella and Shigella were negative, increasing the likelihood of aHUS. On day nine, ADAMTS13 activity was 96.45% with negative inhibitors, excluding TTP. No infections were detected. Clinically, the diagnosis was refined to aHUS. Neurological symptoms showed mild improvement, and supportive therapy was continued while awaiting complement factor and genetic testing (partially limited by financial constraints).

Active TMA and Laboratory-Clinical Dissociation (Day 10-Day 12)

**Figure 4:**
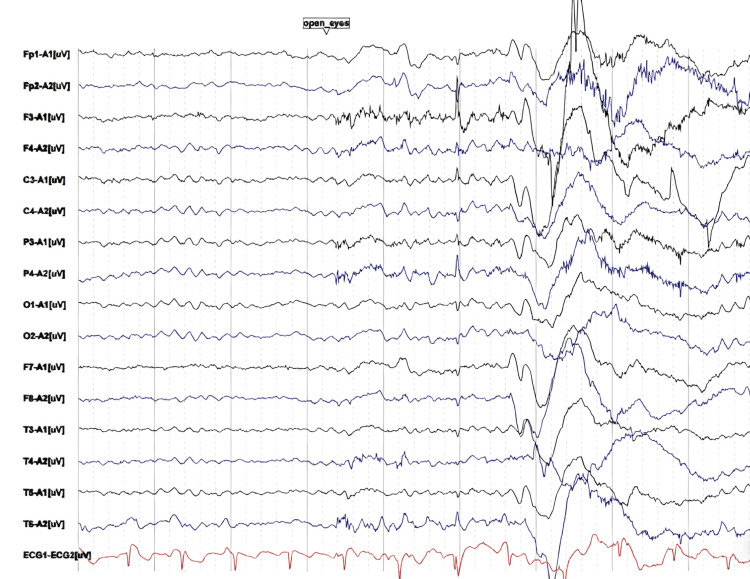
A four-hour continuous EEG demonstrating diffuse slowing of background activity Representative segment of the four-hour continuous EEG showing diffuse slowing of background activity. The recording demonstrates predominant theta and delta waves with attenuation of normal posterior alpha rhythm during the eyes-open state, consistent with diffuse background slowing EEG: electroencephalography

By day 10, mental status had stabilized, but laboratory tests indicated ongoing hemolysis and renal injury (hemoglobin, 84 g/L; LDH, 837.5 U/L; proteinuria, 4+; hematuria, 3+). A 4-hour continuous EEG demonstrated generalized slowing of background activity (Figure 4), indicating dissociation between clinical and biochemical recovery. Continuous EEG monitoring revealed diffuse background slowing, characterized by predominant theta-delta activity and attenuation of the posterior dominant rhythm, consistent with a standardized interpretation of diffuse cerebral dysfunction. On day 11, bilateral calf vein thrombosis was detected, and low-molecular-weight heparin anticoagulation was initiated. Anti-factor H antibody was negative. Emotional and nutritional issues were addressed with antidepressants and appetite stimulants. Nephrology re-evaluation on day 12 confirmed TMA with normal ADAMTS13 and negative pathogen tests, prioritizing aHUS in the differential diagnosis. Renal biopsy was deferred due to thrombocytopenia and anemia.

Recovery and Consolidation Phase (Day 13-Day 15)

By day 13, the patient’s mood and communication had significantly improved, with stable vital signs and resolution of bleeding tendency. LDH decreased to 547.4 U/L and D-dimer to 3.50 mg/L, though 24-hour urinary protein remained elevated (9616.7 mg/24h), indicating delayed renal recovery. Hemoglobin was 75 g/L, with mild hyponatremia and hypoproteinemia. On day 15, diagnostic criteria for HUS were fully met: MAHA (low hemoglobin, high LDH, low haptoglobin, schistocytes >1%), thrombocytopenia, and normal ADAMTS13 (96.45%). Given the exposure to compound chlorzoxazone and repeatedly negative infectious workup, a diagnosis of drug-associated HUS was established. Cerebrospinal fluid testing for the 14-3-3 protein was negative, excluding Creutzfeldt-Jakob disease, and findings were consistent with HUS-related metabolic encephalopathy.

Discharge and Follow-Up Plan (Day 16-Day 17)

**Figure 5:**
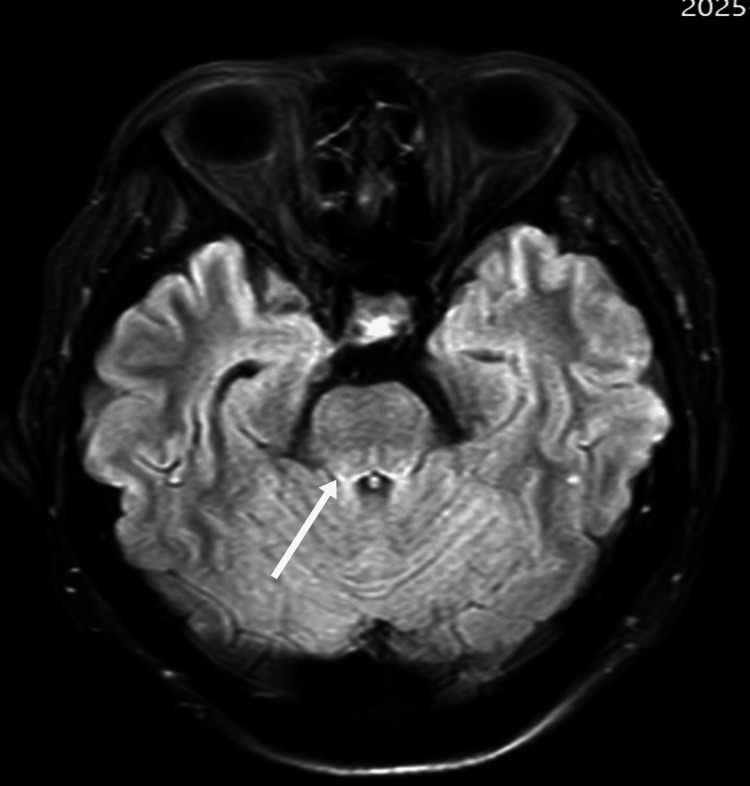
Axial FLAIR MRI of the cerebral peduncle after treatment Follow-up axial T2-FLAIR image demonstrates marked resolution of the previously noted hyperintense lesion in the cerebral peduncle. As indicated by the white arrow, there is a reduction in the abnormal signal of the cerebral peduncle FLAIR: fluid-attenuated inversion recovery; MRI: magnetic resonance imaging

**Figure 6:**
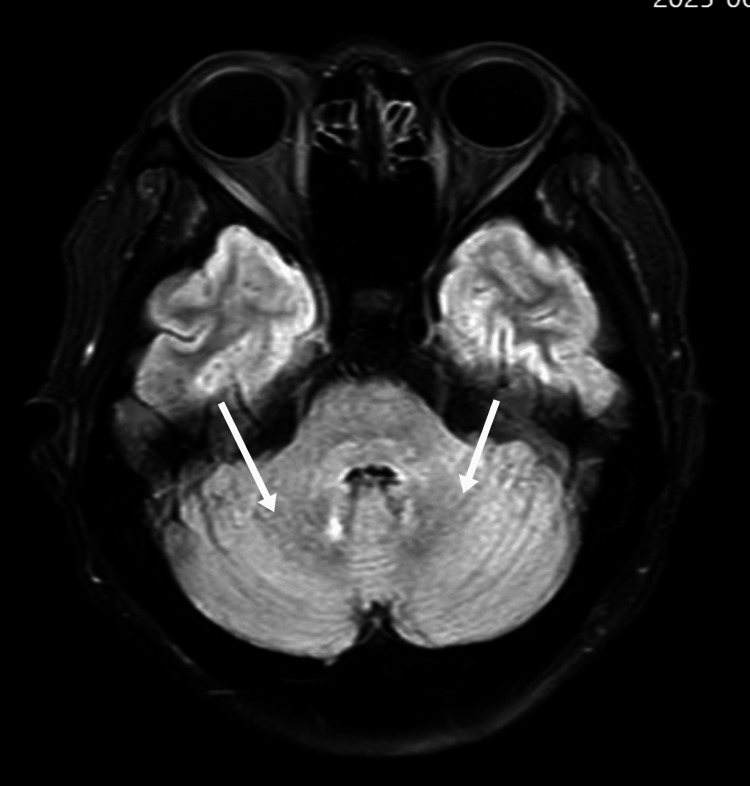
Axial FLAIR MRI of the cerebellum after treatment As indicated by the white arrow, the follow-up axial T2-FLAIR image demonstrates near-complete resolution of the previously observed bilateral hyperintense lesions in the cerebellar dentate nuclei FLAIR: fluid-attenuated inversion recovery; MRI: magnetic resonance imaging

By day 16, the patient was stable and ambulating independently, and neuropsychiatric symptoms had resolved. Hemolysis was controlled, and MRI on day 17 demonstrated resolution of prior lesions in the cerebral peduncle and parietal cortex, with marked reduction of bilateral dentate nucleus abnormalities (Figures 5-6). LDH decreased to 432.7 U/L, with persistent moderate anemia (hemoglobin, 78 g/L) and hypoproteinemia. The final diagnosis was drug-induced HUS. The patient was discharged with recommendations for continued anticoagulation (rivaroxaban), nutritional and metabolic optimization, weekly laboratory monitoring (complete blood count, urinalysis, renal function, hemolysis markers), and multidisciplinary follow-up with neurology, nephrology, and endocrinology. Re-exposure to the suspected drug was strictly contraindicated, and complement-related genetic testing was advised when feasible.

## Discussion

Neurological involvement and clinical significance

Neurological complications are among the most severe manifestations of HUS. Previous studies have reported CNS involvement in approximately 20-50% of cases, presenting as headache, seizures, altered mental status, hemiparesis, ataxia, or even coma [3,5]. A large-scale pediatric study demonstrated that the presence of acute neurological symptoms significantly increased in-hospital mortality (13.9% vs. 1.8%), underscoring the prognostic importance of CNS involvement [6]. Epilepsy is one of the most common neurological manifestations, with an incidence of up to 40% in some typical HUS patients [7].

In this case, early cognitive decline, psychomotor retardation, emotional lability, and reduced speech were noted. MRI showed bilateral cerebellar dentate nucleus and parietal cortical abnormalities, while EEG demonstrated diffuse background slowing, collectively supporting CNS involvement. Initially, these manifestations were misinterpreted as an anxiety state or sequelae of cerebral infarction, leading to delayed recognition of thrombotic microangiopathy. Clinicians should be vigilant for unexplained neuropsychiatric symptoms with proteinuria, hematuria, or coagulation abnormalities, which may indicate HUS [8,9].

Diagnostic differentiation

The diagnostic process in this case evolved progressively. HUS and thrombotic TTP both fall under the TMA spectrum and share overlapping features; however, ADAMTS13 activity serves as a critical discriminator. Severe ADAMTS13 deficiency (<10%) is diagnostic for TTP, whereas normal activity - as seen in this patient (96.45%) - effectively excludes it [10]. Typical HUS is generally associated with Shiga toxin-producing Escherichia coli infection and often presents with diarrhea. The absence of gastrointestinal symptoms and repeatedly negative stool cultures and Shiga toxin assays in this case ruled out STEC-HUS, supporting an atypical form [11]. Persistently low complement C3 levels suggested alternative pathway activation, a finding frequently observed in aHUS, though not entirely specific [12].

Importantly, the patient had recently taken a compound chlorzoxazone preparation. Numerous drugs - such as quinine, cyclosporine, tacrolimus, and gemcitabine - have been implicated in secondary TMAs via immune-mediated or direct endothelial injury mechanisms [13]. Thus, given the negative infectious workup, complement abnormalities, and strong temporal relationship to drug exposure, the diagnosis of drug-induced HUS was deemed most consistent. Even when genetic or complement assays are limited by resource constraints, a structured diagnostic approach - integrating ADAMTS13 testing, infection exclusion, and drug history - can provide reliable evidence for clinical decision-making [14].

Therapeutic management

HUS treatment emphasizes supportive care and trigger removal. For typical HUS, the management focuses on fluid balance, blood pressure control, correction of electrolyte imbalances, and renal replacement therapy when indicated. Routine use of antibiotics or plasma exchange is not recommended unless specific indications exist [15-17].

In contrast, complement inhibition (e.g., eculizumab or ravulizumab) represents the cornerstone of therapy for aHUS, substantially improving renal outcomes and overall survival [18,19]. However, the high cost and limited availability of complement inhibitors restrict their widespread use in many regions, including China [20,21]. In this case, due to financial limitations, complement and genetic analyses were incomplete, and targeted therapy could not be administered. Plasma exchange was not performed, as the patient’s ADAMTS13 activity was normal (96.45%) and her PLASMIC score was low, making immune-mediated TTP unlikely. In accordance with current guidelines, PLEX was therefore not indicated. The patient nevertheless achieved clinical stabilization through intensive supportive care - including fluid and electrolyte management, antihypertensive therapy, anticoagulation, anemia correction, nutritional support, and psychological intervention - which aligns with current evidence-based recommendations [15,16].

Additionally, multidisciplinary team involvement - comprising hematology, nephrology, neurology, and critical care specialists - played a vital role in improving diagnostic efficiency and guiding treatment [22,23]. Platelet transfusion was avoided unless bleeding was life-threatening [24,25]. Early psychological support was also instrumental in reducing anxiety and improving treatment adherence [19].

Laboratory test results

**Figure 7:**
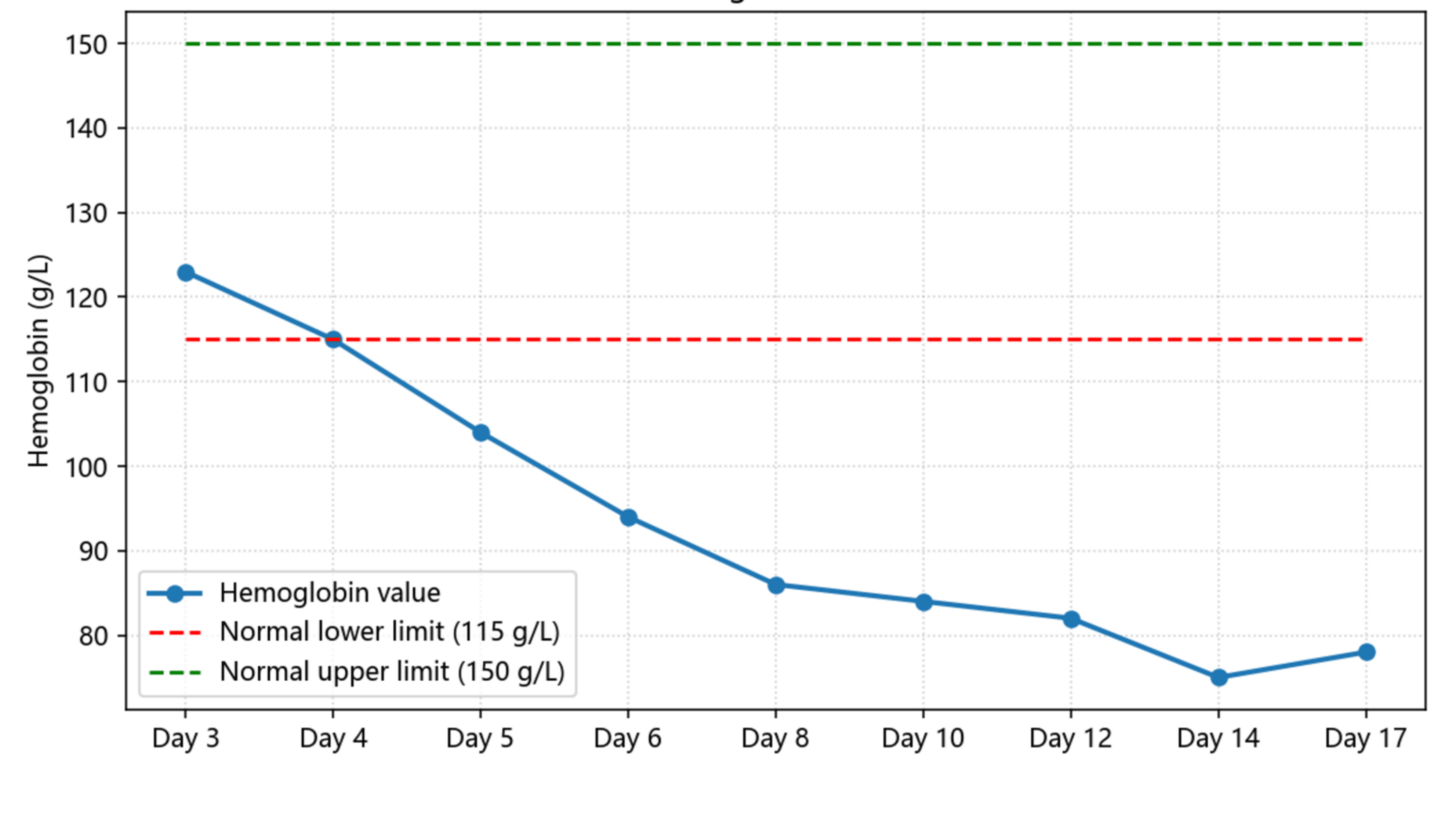
Changes in hemoglobin levels during hospitalization The chart shows the progressive decline and recovery of hemoglobin during hospitalization

**Figure 8:**
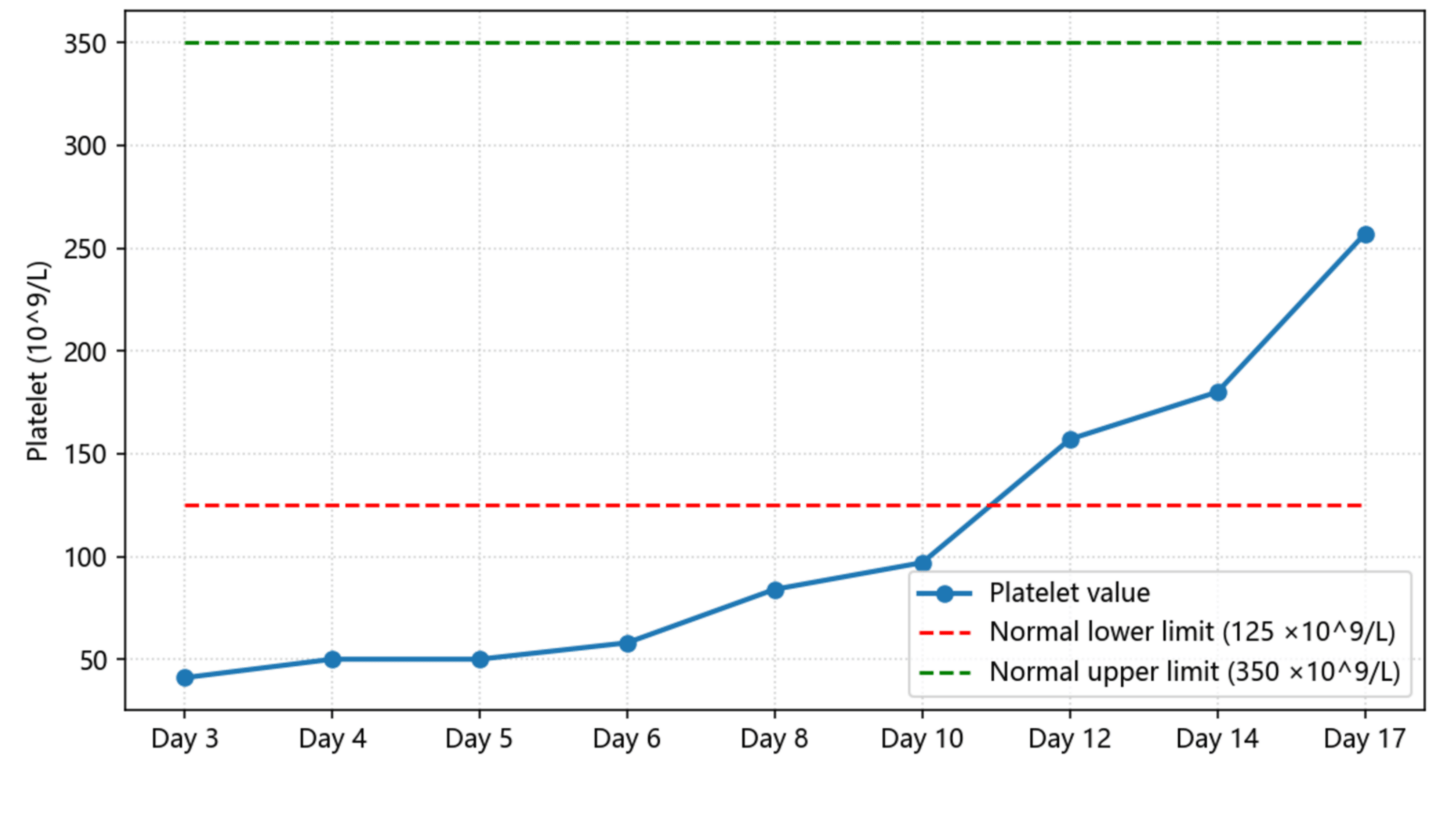
Changes in platelet count during hospitalization The chart illustrates the marked decline in platelet count followed by gradual normalization after treatment, consistent with thrombotic microangiopathy resolution

**Figure 9:**
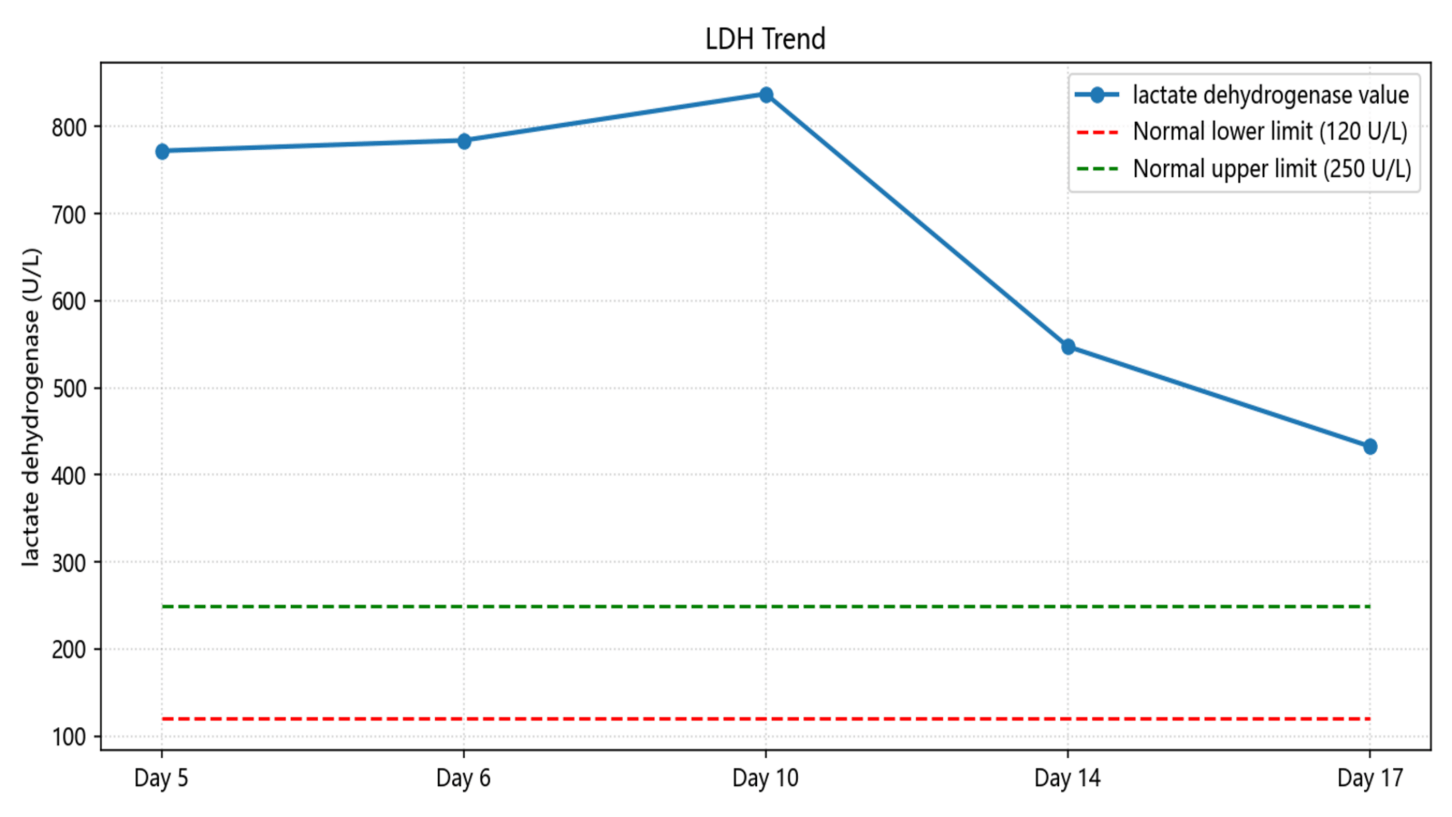
Trends in LDH levels during hospitalization The chart demonstrates the elevation and subsequent reduction of LDH levels, paralleling the activity and remission of hemolysis LDH: lactate dehydrogenase

**Figure 10:**
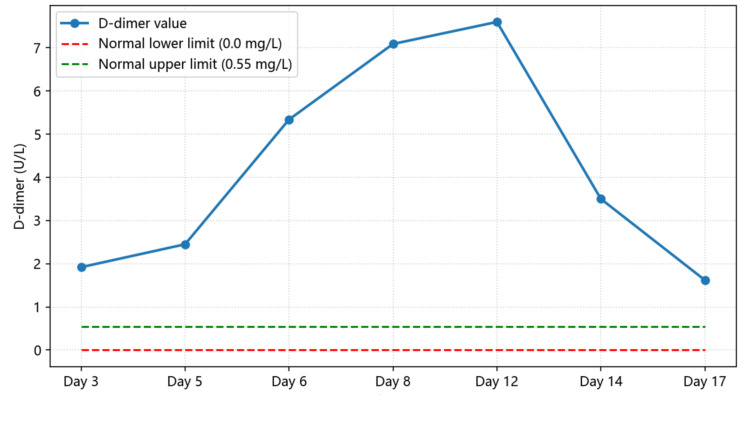
Fluctuations in D-dimer levels during hospitalization The chart depicts changes in D-dimer concentration, indicating activation of the coagulation-fibrinolytic system during the acute phase and its gradual normalization with recovery

In this patient, a systematic diagnostic approach - recognition of the TMA triad, exclusion of TTP (via normal ADAMTS13 activity), and identification of potential drug exposure - led to a final diagnosis of drug-induced atypical HUS. Serial monitoring of key laboratory parameters such as hemoglobin (Figure 7), platelet count (Figure 8), LDH (Figure 9), haptoglobin, and D-dimer (Figure 10) was essential for confirming disease activity and therapeutic response. As part of the chain of evidence, Figures 1-4 in this case clearly demonstrate the dynamic course of the core TMA measures, highlighting the critical importance of continuous and close laboratory monitoring in suspected cases.

Literature review

Pathophysiological Mechanisms

Drug-induced TMA, including HUS, can be broadly categorized into two mechanistic types: immune-mediated and dose- or toxicity-dependent endothelial injury [13]. The immune-mediated form - exemplified by quinine-induced TMA - typically occurs abruptly after drug exposure or re-exposure and is mediated by drug-dependent antibodies targeting platelets, neutrophils, or endothelial cells [25]. In contrast, the toxic form develops subacutely and is dose-dependent, involving direct endothelial damage and vascular dysregulation, as observed with cytotoxic or anti-angiogenic agents [26]. Regardless of the underlying pathway, endothelial injury plays a central role, initiating platelet aggregation, complement activation, and microvascular thrombosis, which culminate in the clinical triad of hemolysis, thrombocytopenia, and AKI. Immediate discontinuation of the causative drug and initiation of intensive supportive care are essential to halt progression and prevent irreversible organ damage. Complement inhibition (e.g., eculizumab) may be considered in refractory or complement-mediated cases.

Common Causative Drugs

Drug-induced HUS/TMA has been reported in association with several therapeutic classes:

Immune-mediated (drug-dependent antibodies): Quinine is the most well-documented trigger, often leading to abrupt and severe presentations that improve upon drug withdrawal but may leave residual renal impairment. Re-exposure typically results in rapid and more severe recurrence [27]. Ticlopidine and clopidogrel have also been implicated through immune mechanisms resembling TTP, necessitating differentiation by ADAMTS13 testing [28].

Dose/toxicity-related endothelial injury: Gemcitabine can cause delayed-onset TMA characterized by progressive hypertension, proteinuria, and renal dysfunction. Prompt discontinuation is mandatory, and complement blockade has shown benefit in refractory cases [29]. Mitomycin C and cisplatin exhibit cumulative dose-dependent endothelial toxicity and poor renal prognosis [28].

Calcineurin and mTOR inhibitors: Cyclosporine and tacrolimus are known to induce post-transplant TMA via endothelial injury and vascular tone imbalance. The management involves dose reduction or substitution, and emerging evidence suggests potential efficacy of eculizumab or belatacept in refractory cases [30].

Anti-vascular endothelial growth factor (VEGF) and anti-angiogenic agents: Bevacizumab and tyrosine kinase inhibitors can cause glomerular microangiopathy (G-TMA) manifesting as hypertension, proteinuria, and renal dysfunction. Pathological findings typically reveal endothelial swelling and capillary thrombosis. Discontinuation is the primary intervention, with occasional benefit from complement inhibition in resistant cases [31].

Chlorzoxazone-Related Evidence and Causality Assessment

In the present case, the patient had been taking a compound chlorzoxazone preparation containing chlorzoxazone and acetaminophen. Although no prior reports directly link this combination to HUS, chlorzoxazone is a well-established in vivo probe for cytochrome P450 2E1 (CYP2E1) activity, and its metabolic activation has been associated with hepatic injury [32]. CYP2E1-mediated metabolism generates excessive reactive oxygen species (ROS), leading to mitochondrial oxidative stress and dysfunction, which can in turn impair hepatic and endothelial integrity [33].

Oxidative stress has been recognized as a major contributor to endothelial dysfunction, compromising vascular defense mechanisms and promoting microvascular injury, a key initiating event in the pathogenesis of HUS [34,35]. Based on this evidence, it is biologically plausible that chlorzoxazone and its reactive metabolites may indirectly trigger endothelial injury through ROS-mediated mechanisms, thereby predisposing to TMA in susceptible individuals. Future in vitro studies assessing endothelial injury after chlorzoxazone exposure could clarify this mechanism. Although CYP2E1-mediated oxidative stress provides a biologically plausible but unproven pathway that could theoretically contribute to endothelial vulnerability, this mechanism remains speculative. Existing data are based on observations of ROS-related hepatic or endothelial stress and do not constitute direct evidence linking chlorzoxazone metabolism to TMA. Therefore, this proposed mechanism should be viewed as hypothesis-generating rather than confirmatory, and further experimental studies would be required to establish any causal role.

Because the compound preparation also contained acetaminophen, we considered whether it might have contributed to the patient’s presentation. However, a review of published case reports, pharmacovigilance data, and mechanistic studies revealed no evidence linking acetaminophen to hemolytic uremic syndrome or other thrombotic microangiopathies. Acetaminophen toxicity is typically limited to hepatic injury at overdose levels and has not been associated with endothelial damage or complement-mediated microvascular pathology. Therefore, its role as a confounder in this case appears unlikely.

To provide an objective estimation of drug causality, the Naranjo Adverse Drug Reaction Assessment Scale was applied. The patient received a total score of 6 (Table 1), which corresponds to a probable association. This classification does not imply definitive causation but rather indicates that the temporal relationship and supporting clinical features are consistent with a drug-related event in the absence of a more likely alternative etiology. It is important to note that the Naranjo scale has inherent limitations when applied to rare conditions such as drug-associated HUS; several scoring items, including rechallenge, dose-response effects, and measurement of drug levels, are typically not feasible or ethically appropriate in this context. Therefore, the score should be interpreted cautiously and integrated with the overall clinical assessment. Further clinical and experimental studies are warranted to elucidate how chlorzoxazone metabolism and oxidative stress contribute to complement activation and endothelial injury. Clinicians should remain cautious when prescribing chlorzoxazone-containing preparations, especially in patients with metabolic comorbidities.

**Table 1:** Naranjo Adverse Drug Reaction Assessment Scale score Naranjo Adverse Drug Reaction Probability Scale categories and interpretation of total scores: scores ≥9 indicate a definite causal relationship between the drug and the adverse reaction, scores of 5-8 indicate a probable relationship, scores of 1-4 indicate a possible relationship, and scores ≤0 indicate a doubtful association [36]

	Evaluation item	Response	Score
1	Are there previous conclusive reports on this adverse drug reaction (ADR)?	No	0
2	Did the adverse event appear after the suspected drug was administered?	Yes	2
3	Did the adverse reaction improve when the drug was discontinued, or a specific antagonist was administered?	Yes	1
4	Did the adverse reaction reappear when the drug was readministered?	Unknown	0
5	Are there alternative causes that could have solely caused the reaction?	No	2
6	Did the reaction reappear when a placebo was given?	Unknown	0
7	Was the drug detected in any body fluid in toxic concentrations?	Unknown	0
8	Was the reaction more severe when the dose was increased, or less severe when the dose was decreased?	Unknown	0
9	Did the patient have a similar reaction to the same or similar drugs in any previous exposure?	No	0
10	Was the adverse event confirmed by any objective evidence?	Yes	1
11	Total score		6

Limitations and perspectives

Although the patient was ultimately diagnosed with drug-associated HUS, this report has certain limitations. Firstly, a renal biopsy was not performed due to thrombocytopenia and patient preference, preventing histopathological confirmation and precise subtyping. Second, complement gene testing was not completed, leaving the possibility of underlying hereditary complement dysregulation unresolved. Finally, long-term follow-up is still needed to evaluate renal and neurological recovery.

These limitations highlight the need for comprehensive diagnostic evaluation - including histopathology and genetic analysis - whenever feasible. Such measures would enhance diagnostic accuracy, facilitate individualized treatment planning, and contribute to a better understanding of the pathophysiological mechanisms underlying drug-induced HUS. To our knowledge, this is the first reported case of compound chlorzoxazone-associated HUS with predominant neuropsychiatric manifestations.

## Conclusions

This report highlights the diagnostic and therapeutic challenges of HUS presenting with acute neuropsychiatric symptoms in an adult patient. HUS can manifest as a multisystem disorder, and when the initial presentation involves cognitive or emotional disturbances, it may easily be mistaken for a primary psychiatric or neurological disease. Clinicians must remain alert to atypical manifestations and consider HUS or TMA when encountering unexplained neurological or mental status changes accompanied by renal or hematologic abnormalities. They should consider drug-associated HUS as a possible cause in similar presentations, while recognizing that causality often cannot be established with certainty. Effective management in our case required comprehensive supportive care and multidisciplinary collaboration, including nephrology, hematology, and neurology input. Even without access to complement inhibitors or plasma exchange, timely supportive treatment and coordinated team care achieved a favorable recovery. This report underscores the importance of maintaining clinical vigilance, performing continuous laboratory surveillance, and pursuing detailed medication history to facilitate early diagnosis and improve outcomes in atypical or drug-related HUS.
